# A phase I trial of weekly gemcitabine and concurrent radiotherapy in patients with locally advanced pancreatic cancer

**DOI:** 10.1038/sj.bjc.6600256

**Published:** 2002-05-03

**Authors:** M Ikeda, S Okada, K Tokuuye, H Ueno, T Okusaka

**Affiliations:** Hepatobiliary and Pancreatic Oncology Division, National Cancer Center Hospital, 5-1-1 Tsukiji, Chuo-ku, Tokyo 104-0045, Japan; Radiation Oncology Division, National Cancer Center Hospital, Tokyo, Japan

**Keywords:** pancreatic cancer, gemcitabine, chemoradiotherapy, CA19-9

## Abstract

This study investigated the maximum-tolerated dose of gemcitabine based on the frequency of dose-limiting toxicities of weekly gemcitabine treatment with concurrent radiotherapy in patients with locally advanced pancreatic cancer. Fifteen patients with locally advanced pancreatic cancer that was histologically confirmed as adenocarcinoma were enrolled in this phase I trial of weekly gemcitabine (150–350 mg m^−2^) with concurrent radiotherapy (50.4 Gy in 28 fractions). Gemcitabine was administered weekly as an intravenous 30-min infusion before radiotherapy for 6 weeks. Three of six patients at the dose of 350 mg m^−2^ of gemicitabine demonstrated dose-limiting toxicities involving neutropenia/ leukocytopenia and elevated transaminase, while nine patients at doses of 150 mg m^−2^ and 250 mg m^−2^ did not demonstrate any sign of dose-limiting toxicity. Of all 15 enrolled patients, six patients (40.0%) showed a partial response. More than 50% reduction of serum carbohydrate antigen 19-9 level was observed in 13 (92.9%) of 14 patients who had pretreatment carbohydrate antigen 19-9 levels of 100 U ml^−1^ or greater. The maximum-tolerated dose of weekly gemcitabine with concurrent radiotherapy was 250 mg m^−2^, and this regimen may have substantial antitumour activity for patients with locally advanced pancreatic cancer. A phase II trial of weekly gemcitabine at the dose of 250 mg m^−2^ with concurrent radiation in patients with locally advanced pancreatic cancer is now underway.

*British Journal of Cancer* (2002) **86**, 1551–1554. DOI: 10.1038/sj/bjc/6600256 www.bjcancer.com

© 2002 Cancer Research UK

## 

Pancreatic cancer (PC) is diagnosed at an advanced stage in most patients, despite recent improvements in diagnostic techniques. Among these patients, roughly half are diagnosed with locally advanced disease radiographically confined to the pancreas and surrounding tissues (Okada, 1999). In the patients with locally advanced PC, prospective randomized trials conducted by Moertel *et al* (1969) and the Gastrointestinal Tumor Study Group (GITSG) (Moertel *et al*, 1982; Gastrointestinal Tumor Study Group, 1988) have demonstrated that the combination of radiotherapy and chemotherapy (chemoradiotherapy; CRT) resulted in significantly better survival than either radiotherapy alone or chemotherapy alone. In patients with resectable PC, the randomised controlled trial conducted by GITSG demonstrated a significant survival advantage for patients receiving adjuvant CRT (Gastrointestinal Tumor Study Group, 1987), but the European Study Group for Pancreatic Cancer (ESPAC) trial failed to show the survival benefit of adjuvant CRT (Neoptolemos *et al*, 2001). Thus, CRT is presently accepted as the standard treatment for locally advanced PC (Okada, 1999), but the role of adjuvant CRT for patients with resectable PC remains controversial. However, optimal CRT regimens have not yet been determined, although various anticancer agents and radiation schedules are being examined in clinical trials.

Gemcitabine is a novel deoxycitidine analogue with a broad spectrum of antitumour activity against a variety of solid tumours including PC (Abratt *et al*, 1994; Casper *et al*, 1994). In patients with advanced PC, gemcitabine demonstrated a greater clinical benefit and survival compared with 5-fluoruracil (Bruiss *et al*, 1997). Gemcitabine has also been shown to be a potent radiosensitizer in human pancreatic and other solid tumour cell lines (Lawrence *et al*, 1996; Shewach and Lawrence, 1996; van Putten *et al*, 2001), suggesting that the combination of radiotherapy and gemcitabine may improve survival in patients with locally advanced PC. Therefore, we conducted a phase I trial to determine the maximum-tolerated dose (MTD) of gemcitabine based on the frequency of dose-limiting toxicities (DLT) of weekly gemcitabine treatment with concurrent radiotherapy in patients with locally advanced PC.

## PATIENTS AND METHODS

### Eligibility

Patients eligible for study entry had histologically or cytologically confirmed locally advanced nonresectable PC. Eligibility criteria were: 20–74 years of age; Karnofsky performance status of 50–100 points; no evidence of distant metastasis, measurable or assessable disease, an estimated life expectancy ⩾8 weeks after study entry; no prior treatment for PC; adequate haematological function (haemoglobin ⩾10 g dl^−1^, leukocytes ⩾4000 mm^3^, neutrophils >2000 mm^3^, and platelets ⩾100 000 mm^3^), adequate hepatic function (serum total bilirubin ⩽2.0 mg dl^−1^ and serum transaminases (glutamic oxaloacetic transaminase (GOT)/glutamic pyruvic transaminase (GPT)) ⩽2.5 times upper normal limit (UNL)), and adequate renal function (serum creatinine within normal limit); written informed consent.

The exclusion criteria were as follows: active infection; severe heart disease; interstitial pneumonitis or pulmonary fibrosis; pleural effusion or ascites; active gastroduodenal ulcer; severe mental disorder, active concomitant malignancy; pregnant and lactating females; females of childbearing age unless using effective contraception; other serious medical conditions.

Ultrasonography, computed tomography of the abdomen, and chest X-ray were performed as pretreatment staging to assess the local extension of the tumour and exclude the presence of distant metastasis. The criteria of computed tomography for the nonresectability of the tumour included tumour encasement of the celiac trunk and/or superior mesenteric artery, and obstruction or bilateral invasion of the portal vein. All patients with obstructive jaundice underwent percutaneous transhepatic or endoscopic retrograde biliary drainage before CRT.

### Treatment schedule

Radiation therapy was delivered through four fields as a single course of 50.4 gray (Gy) in 28 fractions over 5.5 weeks, using 10–14 megavolt photons (MM22, Scanditronix, Uppsala, Sweden). The radiation field included the primary tumour and a margin of 1–3 cm covering the regional lymph nodes, and was defined by treatment-planning computed tomography (GE9800, GE Medical Systems, Milwaukee, WI, USA) obtained 1 or 2 days before initiation of CRT. Lateral treatments were administered together with anteroposterior : posteroanterior (AP : PA) fields so that radiation to the spinal cord could be limited to 40 Gy.

Gemcitabine was administered intravenously over 30 min starting 2 h before radiotherapy weekly for 6 weeks. The scheduled dose of gemcitabine was initially 350 mg m^−2^ per week. However, because of excessive toxicity at this initial dose level, the treatment plan was revised to include lower dose levels of 150 and 250 mg m^−2^ per week. Prophylactic antiemetics were not administered. However, in patients who experienced grade 2 or higher nausea/vomiting, antiemetics including granisetron (Kytril; SmithKline Beecham, Philadelphia, PA, USA) were permitted during subsequent administration of gemcitabine.

Patient cohorts had a minimum of three patients at each dose level. If no DLT was observed in the initial three patients, the dosage was escalated in successive cohorts. If DLT was observed in one or two of the initial three patients, three additional patients were evaluated at that dose level. If only one or two of six patients experienced DLT, dose escalation would continue. However, if three or more patients experienced DLT at a given dose level, then the previous dose level would be considered MTD. DLT was defined as the following manifestations of toxicity observed during CRT or within 2 weeks after completing CRT: grade 3 leukocytopenia and/or neutropenia with high fever, grade 4 haematological toxicities, serum creatinine of ⩾2.0 times UNL, total bilirubin level of ⩾5.0 times UNL, serum GOT/GPT of ⩾10 times UNL, grade 3 or 4 non-haematological toxicities (excluding nausea, vomiting, anorexia, fatigue, constipation, alopecia, and dehydration), two successive weeks' omission of the administration of gemcitabine owing to any toxicity, or treatment periods exceeding 8 weeks.

When DLT or tumour progression was observed during CRT, CRT was stopped. When grade 3 haematological toxicity, serum creatinine of 1.5–2.0 times UNL, total bilirubin level of 3.0–5.0 times UNL, serum GOT/GPT of 5.0–10 times UNL, and/or grade 2 non-haematological toxicity (excluding nausea, vomiting, anorexia, fatigue, constipation, alopecia, and dehydration) were observed, gemcitabine administration was omitted and postponed to the next scheduled treatment day. The radiotherapy was also suspended, and then resumed when toxicities recovered.

### Toxicity and response evaluation

The primary end-point of this trial was to evaluate the frequency of DLT, and the secondary end-point was to evaluate the potential antitumour activity. Treatment related toxicities were assessed using National Cancer Institute – Common Toxicity Criteria version 2.0. During CRT, complete blood count with differential, serum chemistry, and urinalysis were measured at least once a week. Tumour response was evaluated at the completion of CRT and thereafter every 8 weeks until tumour progression, according to the standard World Health Organization criteria (Miller *et al*, 1981). Serum carcinoembryonic antigen levels (CEA) and serum carbohydrate antigen 19-9 levels (CA19-9) were measured monthly by an immunoradiometric assay. This phase I study was approved by the Institutional Review Board of the National Cancer Center.

## RESULTS

### Patients characteristics

Fifteen patients were enrolled in this study from May 2000 to April 2001 at the National Cancer Center Hospital, Tokyo, Japan. The characteristics of the patients are listed in Table 1

**Table 1:**
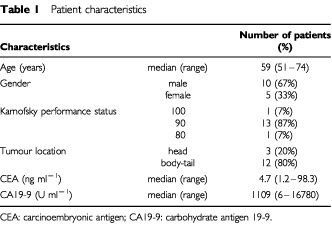
Patient characteristics

. The median age was 59 (range: 51–74) years. Karnofsky performance status was 100 in one patient (7%), 90 in 13 (87%), and 80 in 1 (7%). The median serum CA19-9 level is 1109 (range: 6–16780). Patients were treated with radiation and concurrent weekly gemcitabine over three dose levels, as listed in Table 2

**Table 2:**
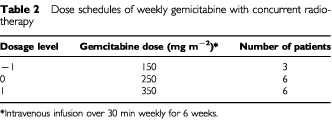
Dose schedules of weekly gemicitabine with concurrent radiotherapy

.

### Toxicity

The toxicities observed in the 15 enrolled patients are listed in Table 3

**Table 3:**
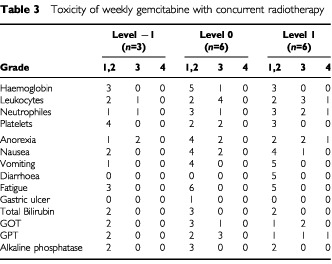
Toxicity of weekly gemcitabine with concurrent radiotherapy

. There was no treatment-related death during this study. Three of six patients treated at the gemcitabine 350 mg m^−2^ dose level experienced DLT; one patient developed grade 4 leukocytopenia/neutropenia, a second developed grade 4 serum GPT increase, and a third required two successive weeks' omission of gemcitabine administration due to serum GOT and GPT increase. Moreover, before DLT was observed in level 1, 8 of 31 planned administrations were omitted due to adverse effects including leukocytopenia, neutropenia, GOT/GPT increase, and severe fatigue. Because the DLTs were observed in three of six patients at the first dose level (350 mg m^−2^), the protocol was revised and subsequent patients were enrolled at lower dose levels (gemcitabine dose level −1: 150 mg m^−2^, level 0: 250 mg m^−2^). Six patients were enrolled in level 0 to evaluate the frequency of DLT more accurately, although no DLT was observed in the initial three patients. One of 18 planned administrations at level −1 and 4 of 36 administrations at level 0 were omitted due to adverse effects, including grade 3 leukocytopenia, grade 3 neutropenia, grade 3 thrombocytopenia, and grade 3 GOT/GPT increase which did not exceed 10 times UNL. However, these toxicities were mild and transient, and all patients treated at level −1 or level 0 completed the scheduled course of CRT without DLT. Gastric ulcer with epigastralgia was observed in one patient (level 0) 1 week after CRT, but the patient recovered with medical treatment using omeprazole (Omepral; Astra Zeneca, Sweden). Thus, weekly gemcitabine at a 150 mg m^−2^ or a 250 mg m^−2^ dose was considered well tolerated.

### Response

Six patients (level 1: 4 patients, level −1: 0 patients, level 0: 2 patients) achieved partial response, giving an overall response rate of 40.0% (95% confidence interval, 15.2–64.8%), and the mean duration of response was 7.5 months (range: 3.3–12.3 months). The remaining nine patients demonstrated stable disease. After the completion of CRT, the serum CA19-9 level was reduced more than 50% in 13 (92.9%) of 14 patients who had shown a pretreatment level of 100 U ml^−1^ or greater, and the serum CEA level was reduced more than 50% in four (80.0%) of five patients who had a pretreatment level of 10 ng ml^−1^ or greater (Table 4

**Table 4:**
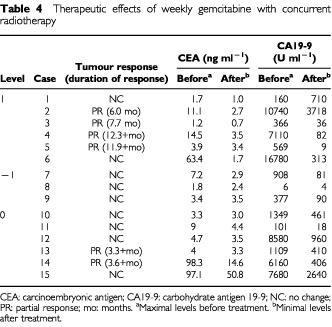
Therapeutic effects of weekly gemcitabine with concurrent radiotherapy

).

## DISCUSSION

Currently, concomitant external beam radiotherapy and chemotherapy has been accepted as the standard therapy for patients with locally advanced nonresectable PC (Okada, 1999), because randomized trials have demonstrated improved survival with CRT compared with that by either radiation alone or chemotherapy alone (Moertel *et al*, 1969, 1981; Gastrointestinal Tumor Study Group, 1988), although the role of adjuvant CRT for patients with resectable PC remains controversial. To intensify the treatment efficacy, various anticancer agents, and radiation schedules are being examined in clinical trials. However, to date, optimal CRT regimens have not yet been determined.

Gemcitabine produced significantly better clinical benefit (decreased pain, improved performance status and/or weight gain) (response rate, 23.8% *vs* 4.8%) and survival advantage (median survival, 5.6 *vs* 4.4 months) compared with bolus 5-fluorouracil in a phase III trial (Burris *et al*, 1997), and the similar effects on disease-related symptoms were documented in patients with 5-fluorouracil-refractory PC (Rothenberg *et al*, 1996). Moreover, in over 3000 patients with advanced PC treated with gemcitabine, notable disease-related symptom improvement (response rate, 18.4%) and survival (median survival, 4.8 months) were also seen (Storniolo *et al*, 1999). Therefore, gemcitabine has recently been accepted as the first-line chemotherapy for advanced PC, particularly in Western countries. In the majority of reported clinical trials including these trials, gemcitabine was administered once weekly. Gemcitabine has also been demonstrated *in vitro* and *in vivo* to enhance the cytotoxic activity of radiation (Lawrence *et al*, 1996; Shewach and Lawrence, 1996), although the precise mechanism of radiosensitization remains unknown (van Putten *et al*, 2001). Some clinical trials of gemcitabine and concurrent radiotherapy have been reported (Blackstock *et al*, 1999; Talamonti *et al*, 2000), but the optimal dosage for a weekly gemcitabine schedule has not yet been elucidated. Although it has been reported that a twice-weekly schedule of gemcitabine is superior to a once-weekly schedule of gemcitabine for radiosensitization (Blackstock *et al*, 1999), the dose of gemcitabine was much lower (40 mg m^−2^, twice-weekly) than that used for systemic chemotherapy. In patients with locally advanced PC treated with CRT, it is important to enhance the local control and simultaneously reduce the risk of distant metastases. Therefore, this phase I trial was designed to determine the MTD of weekly gemcitabine with concurrent radiation in patients with locally advanced PC. Eligibility criteria of this trial included Karnofsky performance status of 50–100 points. However, all enrolled patients had the Karnofsky performance status of 80 or above, because the patients with poor performance status (e.g. Karnofsky performance status of less than 70) were considered to be intolerable to CRT for fear of the treatment-related toxicities such as fatigue.

We expected that the dose of weekly gemcitabine with concurrent radiotherapy would be close to 1000 mg m^−2^, which is the standard dose for weekly gemcitabine administration for PC. However, DLTs involving neutropenia/leukocytopenia and elevated transaminase were observed in three of six patients at the first dose level (350 mg m^−2^), and the protocol was therefore revised to include lower dose levels (150 and 250 mg m^−2^ per week). All nine patients treated at the dose of 150 or 250 mg m^−2^ per week of gemcitabine completed the scheduled course of CRT without DLT. In this CRT, the most common toxicities were leukocyotopenia and/or neutropenia. However, these toxicities were mild and transient at level −1 and level 0, and all patients recovered within a week without any specific treatment for these toxicities. Anorexia and/or nausea observed on the day of gemcitabine administration were the major non-haematological toxicities. However, these toxicities were relieved by prophylactic use of granisetron on subsequent administration of gemcitabine. Serum GOT/GPT increase observed at level 0 (250 mg m^−2^), which was another major non-haematological toxicity, was also mild and transient. Therefore, weekly gemcitabine at a dose of 150 mg m^−2^ or 250 mg m^−2^ was considered well tolerated, and the MTD was determined to be 250 mg m^−2^.

With regard to antitumour activity of weekly gemcitabine with concurrent radiation, 6 out of 15 patients achieved partial response, giving an overall response rate of 40.0% (95% confidence interval, 15.2–64.8%). Moreover, the serum CA19-9 level was reduced more than 50% after the completion of CRT in 13 (92.9%) of 14 patients with a pretreatment level of 100 U ml^−1^ or greater. These results are favourable compared with those of patients treated with CRT at other treatment schedules (Moertel *et al*, 1969, 1981; Gastrointestinal Tumor Study Group, 1988; Ishii *et al*, 1997; Blackstock *et al*, 1999; Talamonti *et al*, 2000; Okusaka *et al*, 2001). To date, however, there are no data on overall survival and progression-free survival because of the short follow-up periods. Phase II trial is required to clarify the antitumour activity and the effect of weekly gemcitabine with concurrent radiation on survival.

In conclusion, the MTD of weekly gemcitabine with concurrent radiotherapy was 250 mg m^−2^, and this regimen may have substantial antitumour activity for patients with locally advanced PC. A phase II trial of weekly gemcitabine at a dose of 250 mg m^−2^ with concurrent radiation in patients with locally advanced PC is now underway.
